# Cascade contour-enhanced panoptic segmentation for robotic vision perception

**DOI:** 10.3389/fnbot.2024.1489021

**Published:** 2024-10-21

**Authors:** Yue Xu, Runze Liu, Dongchen Zhu, Lili Chen, Xiaolin Zhang, Jiamao Li

**Affiliations:** ^1^Shanghai Institute of Microsystem and Information Technology, Chinese Academy of Sciences, Shanghai, China; ^2^School of Information Science and Technology, ShanghaiTech University, Shanghai, China; ^3^University of Chinese Academy of Sciences, Beijing, China

**Keywords:** robot vision, panoptic segmentation, panoptic contour detection, structure perception, cascade, feature enhancement, visual pathway

## Abstract

Panoptic segmentation plays a crucial role in enabling robots to comprehend their surroundings, providing fine-grained scene understanding information for robots' intelligent tasks. Although existing methods have made some progress, they are prone to fail in areas with weak textures, small objects, etc. Inspired by biological vision research, we propose a cascaded contour-enhanced panoptic segmentation network called CCPSNet, attempting to enhance the discriminability of instances through structural knowledge. To acquire the scene structure, a cascade contour detection stream is designed, which extracts comprehensive scene contours using channel regulation structural perception module and coarse-to-fine cascade strategy. Furthermore, the contour-guided multi-scale feature enhancement stream is developed to boost the discrimination ability for small objects and weak textures. The stream integrates contour information and multi-scale context features through structural-aware feature modulation module and inverse aggregation technique. Experimental results show that our method improves accuracy on the Cityscapes (61.2 PQ) and COCO (43.5 PQ) datasets while also demonstrating robustness in challenging simulated real-world complex scenarios faced by robots, such as dirty cameras and rainy conditions. The proposed network promises to help the robot perceive the real scene. In future work, an unsupervised training strategy for the network could be explored to reduce the training cost.

## 1 Introduction

In recent years, camera-based perception systems have been widely used in various kinds of robots, which brings the need for image-based scene understanding algorithms. For robots, it is not only necessary to recognize the semantic information in the scene, but also to distinguish different instances, which is of great significance for robots to navigate, interact and execute tasks in complex environments. Specifically, robots need the help of computer vision techniques to identify obstacles in the scene, identify users, find targets, etc.

**Figure 1:**
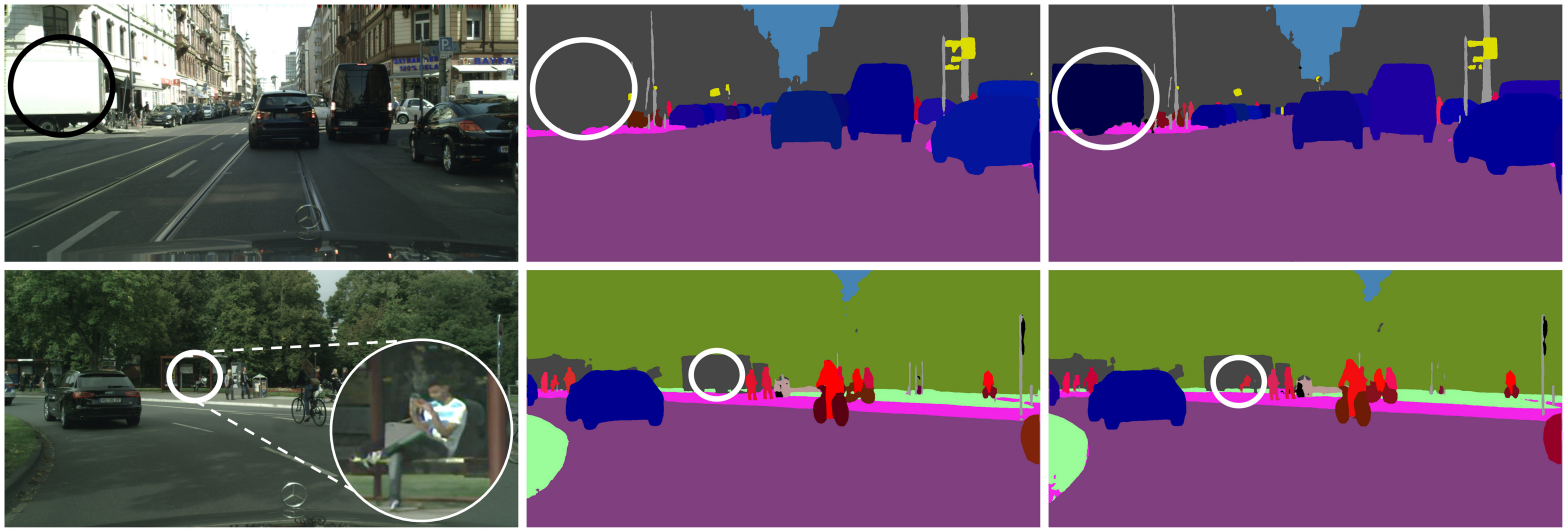
Visualization examples of misclassification and missing detection. **Left**: input image; **middle**: UPSNet results, misclassification of the track and missing detection of the human; **right**: our results.

In order to meet this demand, semantic segmentation (Zhang et al., 2022; Ye et al., 2023; Zhang et al., 2023) and object detection (Liu and Stathaki, 2018; He et al., 2017) tasks have been proposed in the field of computer vision, which are used to identify the semantic information of the pixels in the scene and distinguish the information of different instances in the image respectively. Until Kirillov et al. (2019a) proposed the panoptic segmentation task, which unifies the above two tasks. This new task is dedicated to identifying each pixel's semantic and instance ID in the input image. This task has gained significant attention in the field of scene understanding due to its precise definition. It is a valuable tool for autonomous driving and industrial robotics applications. There are currently three categories of deep learning-based panoptic segmentation methods based on the instance mask generation approach: top-down (Mask R-CNN based), bottom-up (DeepLab based), and transformer-based (DETR based). These methods have shown promising progress on the datasets. However, misclassification of textureless regions and missing detection of small targets remain to be solved. For example, the white truck was wrongly identified as a building because it lacks texture and has the same color as the building behind it. Additionally, the person in the distance was not detected by the current algorithm due to their small size, as shown in Figure 1.

The lack of contour perception may cause the problems above, according to the perceptual theory of biological vision. Research (Zhou et al., 2000) indicated that contour detection plays a crucial role in processing scene data in a monkey's visual cortex. The studies suggest that about 18% of the cells in area V1 and over 50% in V2 and V4 on the cerebral cortex are dedicated to processing contour-related information. It is well verified by the previous semantic segmentation that introduced edge detection as an auxiliary task. Gated-SCNN (Takikawa et al., 2019), DecoupleSegNets (Li et al., 2020), and RPCNet (Zhen et al., 2020) demonstrated the importance of contours in scene recognition by introducing semantic edges to improve semantic segmentation performance. In the panoptic segmentation task, some previous studies (Xu et al., 2021; Chang et al., 2023) have explored contour detection as a separate component in their work. CAPSNet (Xu et al., 2021) was the first work that introduced a contour branch to guide feature extraction and explicit contour supervision on the result of panoptic segmentation to improve the network's understanding of the structure. However, this method did not incorporate contour information into the panoptic segmentation process. SE-PSNet (Chang et al., 2023) adopted a similar structure and used contour as auxiliary information to enhance instance segmentation, but did not contribute to semantic segmentation.

In this study, we introduce the Cascade Contour-enhanced Panoptic Segmentation Network (CCPSNet), a novel approach designed to fully embrace structural knowledge to improve the detection of small targets and the semantic recognition of weak texture areas. Our method employs a cascading strategy to adaptively refine multi-scale structural contour details, thereby facilitating more precise detection of contours in areas lacking texture or containing small objects. Furthermore, we present a contour-guided multi-scale feature enhancement stream that integrates panoptic contours and multi-scale features to refine segmentation features and calibrates the perceptual field using structural-aware feature modulation module(SFMM), thereby enhancing segmentation accuracy. The key contributions of our work are as follows:

- We propose a cascade contour-enhanced panoptic segmentation network, which effectively delves into the comprehensive structural knowledge using panoptic contour detection and corresponding features, thereby improving the robot vision's perception ability in challenging complex areas.
- We develop a cascaded contour detection stream for the panoptic segmentation network, which aims to extract scene structural information by a feature channel regulation module and cascade strategy.
- We design a contour-guided multi-scale feature enhancement stream that incorporates contour information and contextual features to enhance the feature learning of areas with small objects and weak textures.
- Extensive experiments on the Cityscapes and COCO datasets substantiate the robustness and superiority of our proposed network compared to existing methods.

## 2 Related work

In this section, we review the progress of research on deep learning-based panoptic segmentation algorithms and contour detection in deep learning.

### 2.1 Deep learning-based panoptic segmentation

#### 2.1.1 Top-down

Due to the exceptional performance of Mask R-CNN (He et al., 2017) on the instance segmentation task, this type of approach combines semantic segmentation branches with instance segmentation outcomes to generate panoptic segmentation results. The Panoptic-FPN was proposed by Kirillov, which utilized semantic segmentation with a shared Feature Pyramid Network backbone. Since then, many studies (Chen Y. et al., 2020; Li et al., 2019; Liu et al., 2019; Xiong et al., 2019) have expanded upon this approach. UPSNet (Xiong et al., 2019) introduced a parameter-free panoptic head that predicts the final panoptic segmentation via pixel-wise classification, the number of classes per image of which could vary. BANet (Chen Y. et al., 2020) exploits the complementary relationship between semantics and instances to design Semantic-to-Instance module and Instance-to-semantic module to improve the performance. EfficientPS (Mohan and Valada, 2021) is currently the most effective technique in top-down methods. It involves creating a new backbone network and implementing a 2-way FPN while maintaining the core structure of the Mask R-CNN component.

Based on the above studies, CAPSNet (Xu et al., 2021) pioneered the idea of enhancing the network's ability to perceive structures by introducing panoptic contour-aware branches. Then SE-PSNet (Chang et al., 2023) introduced the contour-based enhancement features into different predicted heads. While the previous methods use contour perception to aid in the understanding of structural information within an image, the design of this approach is relatively simple and may struggle with small targets. To address this issue, we introduce a new cascaded panoptic contour detection head to improve detail awareness and a contour attention feature enhancement module to enhance feature expression. These improvements should enhance the overall performance of the system.

#### 2.1.2 Bottom-up

In contrast to the above approaches that use instance partitioning as the core of the network, many studies (Chen et al., 2017; Yang et al., 2019; Cheng et al., 2020; Gao et al., 2019; Wang H. et al., 2020; Sun et al., 2023) that focus more on semantic segmentation and cluster the results to generate instance segmentation results. They adopt models such as DeepLab (Chen et al., 2017), a semantic segmentation model that uses an encoder-decoder architecture with an atrous convolution structure as the backbone to generate semantic segmentation results while generating instance segmentation results through a bottom-up approach. Panoptic-DeepLab (Cheng et al., 2020) adopts the dual-ASPP and dual-decoder structures specific to semantic and instance segmentation, respectively. Deeper-Lab (Yang et al., 2019) uses bounding box and center point to generate the instance mask and SSAP (Gao et al., 2019) proposes a pixel-pair affinity pyramid to predict the instance by computing the probability that two pixels belong to the same instance.

#### 2.1.3 Transformer based

The DEtection TRansformer (DETR) has been proposed by Carion et al. (2020) as a successful application of the Transformer method, commonly used in NLP, for image detection tasks. Many networks (Wang et al., 2021; Yu et al., 2022a,b) have subsequently emerged that employ the self-attention module. Max-DeepLab (Wang et al., 2021) introduces mask transformer to predict class-labeled masks directly, while training with panoptic quality inspired loss via bipartite matching to improve Axial-DeepLab (Wang H. et al., 2020)'s performance on highly deformable objects, or nearby objects with close centers. Building on this work, CMT-DeepLab (Yu et al., 2022a) composes the process of assigning pixels to the clusters by feature affinity and updating the cluster centers and pixel features as Clustering Mask Transformer. kMaX-DeepLab (Yu et al., 2022b) draws on the k-means clustering algorithm and redesigns the cross-attention mechanism by introducing the relationship between pixels and object queries. The above model greatly simplifies the process of panoptic segmentation and has more powerful feature learning capability to improve the performance effectively. However, they require huge computility is unsatisfactory.

### 2.2 Contour detection in deep learning

The edge detection task is a fundamental task in computer vision, and this task has also seen new advances through deep learning this year. Among them, HED (Xie and Tu, 2015) improves the performance by using a pattern of fusion of multiple layers. Meanwhile, edges also play an important role in the segmentation task. Nvidia (Takikawa et al., 2019) proposed to assist segmentation through contours. CAPSNet (Xu et al., 2021) is the first model that proposes to introduce panoptic segmentation contours into the panoptic task. SE-PSNet (Chang et al., 2023) assists panoptic segmentation according to semantic contours and instance contours, respectively.

All of the above methods use contouring as an auxiliary task, and for the first time, our model incorporates panoptic segmentation contouring results into the prediction process.

### 2.3 Attention model in deep learning

Many previous works have demonstrated the outstanding performance of attention mechanisms on various tasks such as object detection (Alazeb et al., 2024), autonomous driving (Yang et al., 2023), saliency prediction (Min et al., 2020), and fixation prediction (Min et al., 2016). Min et al. (2016) utilizes canonical correlation analysis to identify the most relevant audio features. It construct visual attention models through spatial attention and temporal attention to predict fixation points. The moving sound target is located using cross-modal kernel canonical correlation analysis. Min et al. (2020) introduces a two-stage adaptive audiovisual saliency fusion method to complete the saliency prediction. Axial-DeepLab (Wang H. et al., 2020) is a fully attentional network with novel position-sensitive axial-attention layers that combine self-attention for non-local interactions with positional sensitivity. The deletion of the object detection branch leads to those methods being more efficient rather than more effective.

In this paper, we use the attention mechanism to enhance structure perception. The expression of the contour on the feature channel is guided by the attention in the cascaded contour detection stream, and the panoptic contour is used as the input to generate spatial attention to assist the final panoptic segmentation in the contour-guided multi-scale feature enhancement stream.

## 3 Methodology

**Figure 2:**
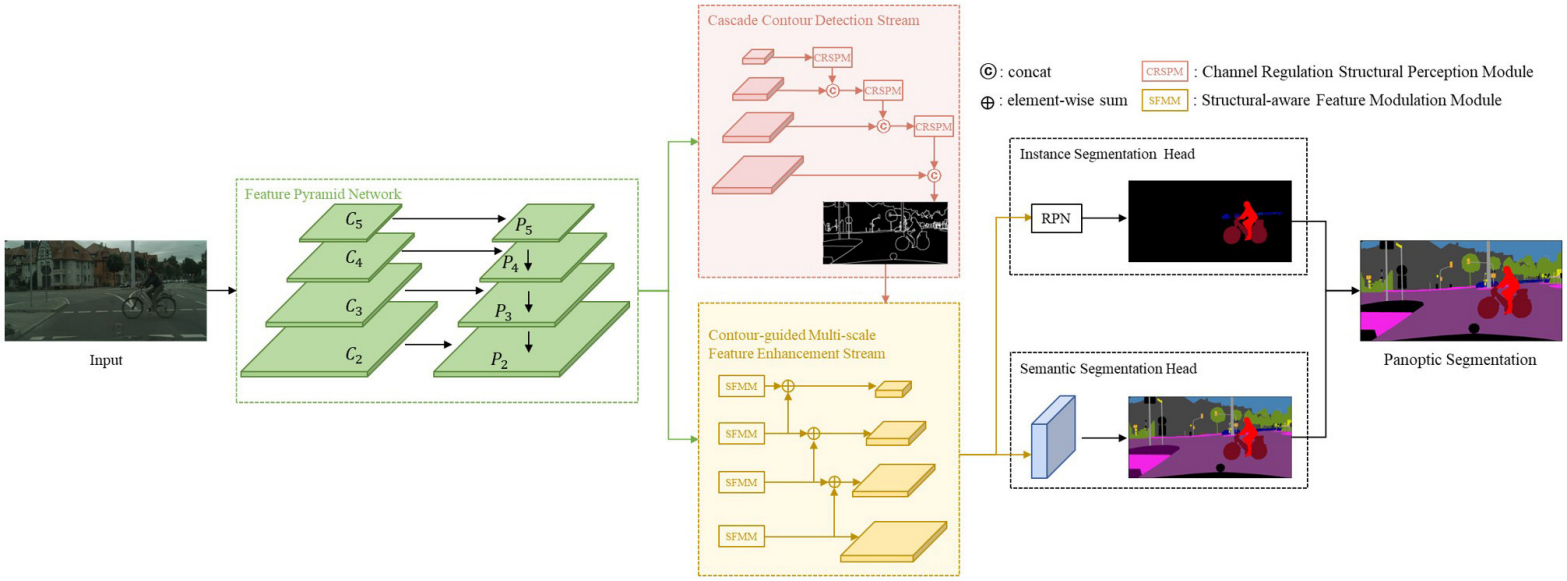
Illustration of the proposed CCPSNet.

In this section, we provide a detailed overview of our proposed network, illustrated in Figure 2. The network follows a top-down, which employs a shared ResNet backbone with a Feature Pyramid Network (FPN). Our network has four major components: (1) Cascade contour detection stream. (2) Contour-guided multi-scale feature enhancement stream to enhance feature expression on the backbone. (3) The instance segmentation head provides the instance segmentation predictions. (4) The semantic segmentation head predicts semantic results. This section provides the details of those.

### 3.1 Cascade contour detection stream

Inspired by Gated-SCNN (Takikawa et al., 2019) and RPCNet (Zhen et al., 2020) that introduce the edge detection to aid in semantic segmentation, contours are critical clues in segmentation task. In order to outline every object or background in the scene, panoptic segmentation contour is an amalgamation of semantic segmentation contour and instance segmentation contour. Particularly, for an image *I*, it's panoptic segmentation contour label *C*_*I*_ = *C*_*s*_∪*C*_*t*_. Here, *C*_*s*_ is the semantic contour for stuff categories and *C*_*t*_ is the instance contour for *things* label. In terms of the truth result of the panoptic segmentation ground truth *P*, specifically for a pixel *P*_*p*_, whenever any of the 8 pixels surrounding this pixel point has a different semantic or instance ID with it, we consider it to be a panoptic contour pixel. Since the results are colored by category and instance, different categories or different objects id in the same category will be colored differently. Thus Laplace convolution can be used to obtain a panoptic segmentation contour.


(1) $$\begin{array}{l} C_{P_{p}}=1\;\forall P_{p}\in P,\;s.t.\;lapulation\left(P_{p}\right)\neq 0. \end{array}$$

**Figure 3:**
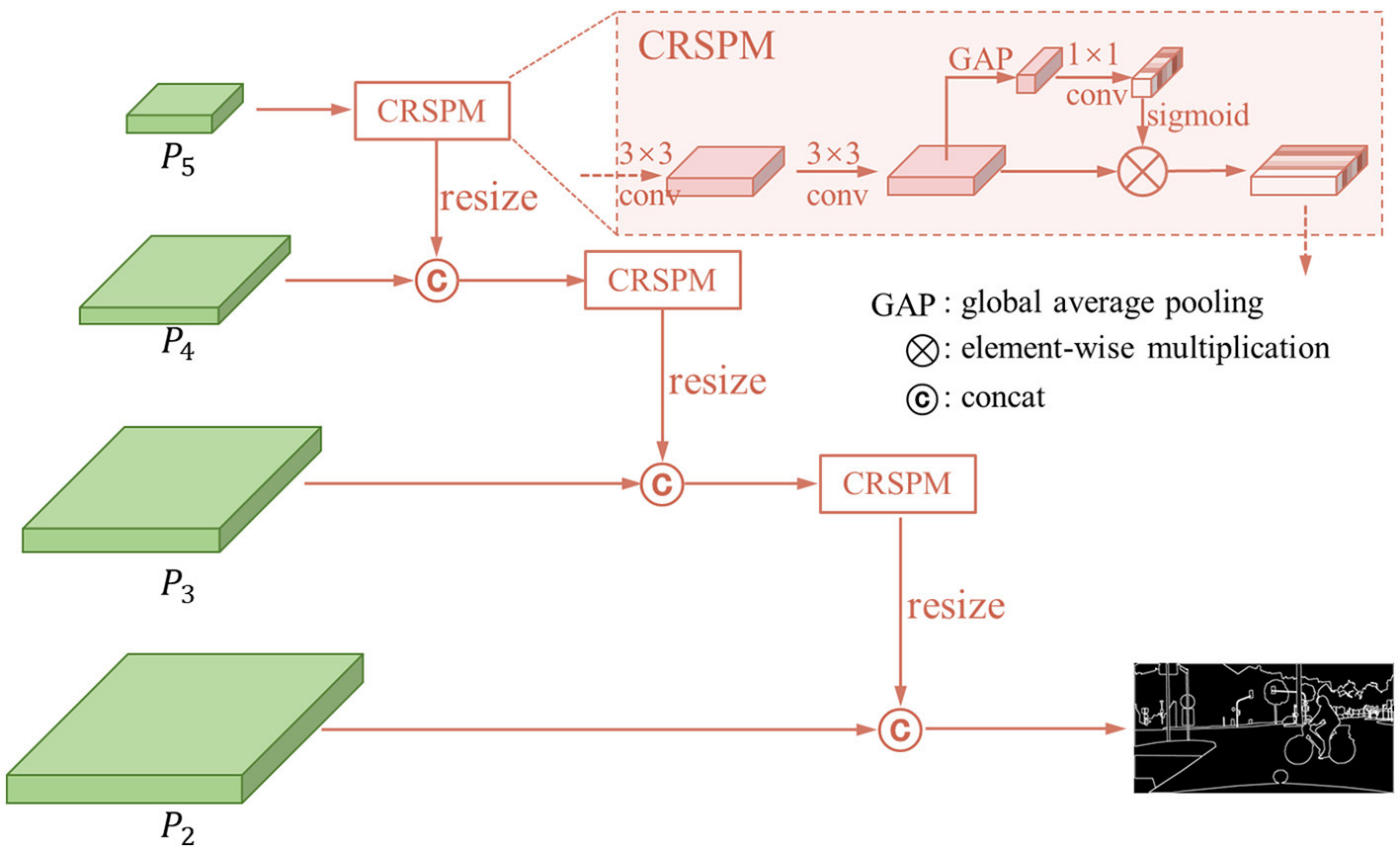
The structure of cascade contour detection stream.

In order to get the contour, we design a cascade contour detection stream. The structure is shown in Figure 3. We introduce the features*P*_2_−*P*_5_ obtained from FPN into the channel regulation structural perception module (CRSPM) to obtain the contour features of the corresponding layers. To begin with, we apply a 3 × 3 convolution to convert the features into contour features. Subsequently, considering the variability of the activation patterns of different convolution kernels, we generate weights on channels to regulate feature volume, which aims to enhance structural information and suppress negative impact information. This module is primarily built through global average pooling and fully connected layers, which can be represented by the following formula:


(2) $$\begin{array}{l} P=f\left(P_{i}\right)⊗g\left(GAP\left(f\left(P_{i}\right)\right)\right) \end{array}$$

In which, *f*() represents the two 3 × 3 convolution layers, *GAP*() represents the global average pooling, and *g*() is the 1 × 1 convolution. By this way, we retain the ability to perceive the texture while picking out the channels that are responsive to the contours. In order to maintain the overall information on the large scale and avoid the misclassification caused by the texture difference inside the structure, the large-scale and small-scale features are fused by concatenate operation. From *P*_5_ to *P*_2_, we apply coarse-to-fine cascade aggregation to get the contour feature and predict the panoptic segmentation contour.

Due to the distribution imbalance of the number of contour and non-contour pixels, we adopt the class-balancing cross-entropy loss function *L*_*c*_ following the HED (Xie and Tu, 2015) as the contour loss. Equation 1 provides its formalization. In which α is the class-balancing weight on a per-pixel term basis. *C* denotes the ground truth of panoptic contour, and Ĉ denotes the predicted panoptic contour. *C*_−_ denotes the contour ground truth label sets.


(3) $$\begin{array}{c} L_{c}=-\alpha Clog(sigmoid(\hat{C}))-(1-\alpha )(1-C)log(1-sigmoid(\hat{C}));\\ \alpha =\frac{C_{-}}{C} \end{array}$$

### 3.2 Contour-guided multi-scale feature enhancement stream

**Figure 4:**
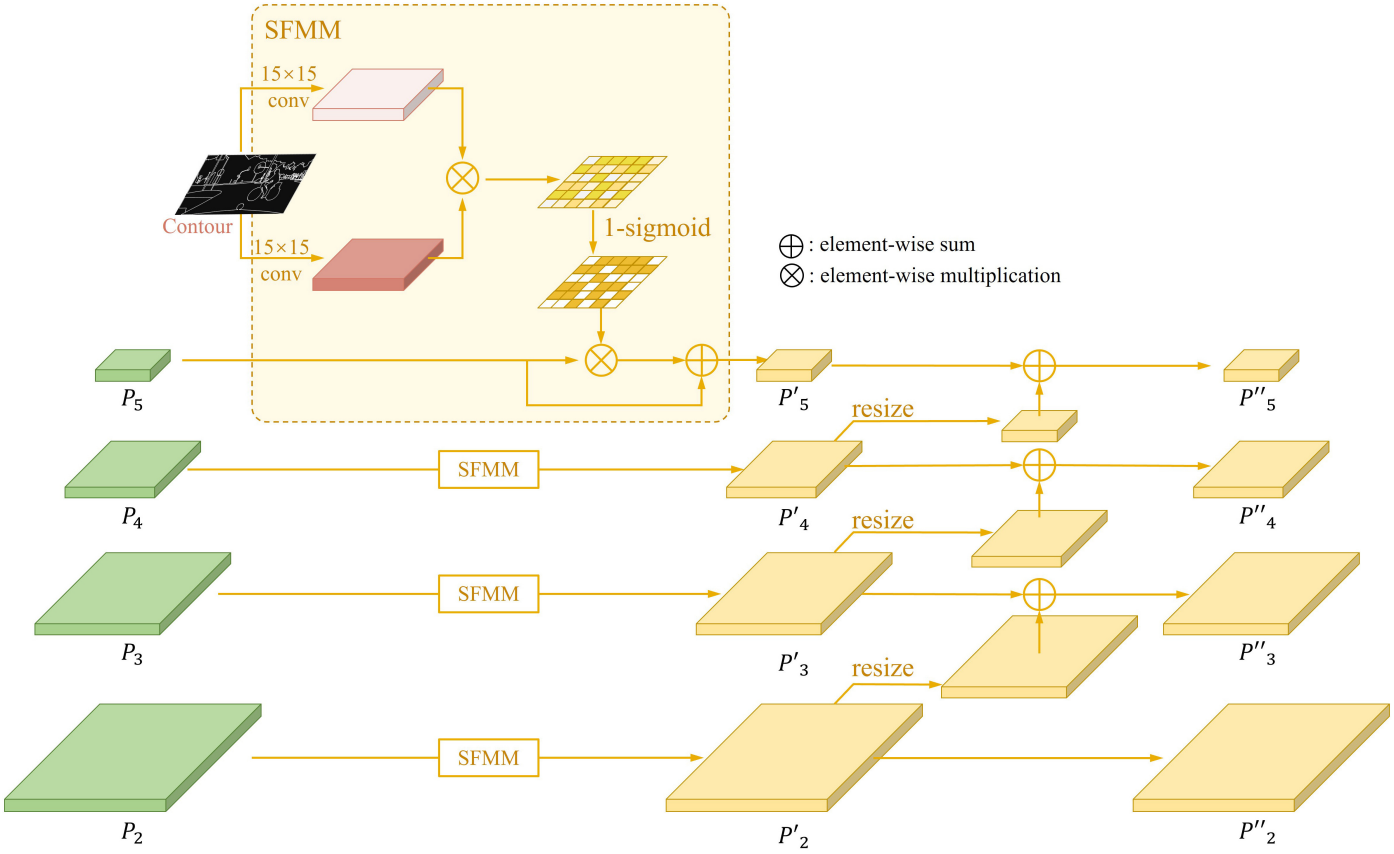
The structure of contour-guided multi-scale feature enhancement stream.

In the process of extracting features from the backbone, a lot of detailed information is lost due to multiple down-sampling and pooling operations, which leads to the mission detection of small objects. To address this issue, we proposed structural-aware feature modulation module (SFMM) and inverse aggregation operation, which augments the features by taking the panoptic contours to generate attention at the spatial scale and fuses with features of different scales. This module helps incorporate structured information into features at all scales to aid in learning small object features. Its structure is shown in Figure 4. Here, we give a detailed formulation for this process.

Given an input feature map $P_{i}\in {\mathbb{R}}^{N\times W_{i}\times H_{i}}$ from the *i*-th scale FPN branch. The contour was resized to same scale with this feature map $C_{i}\in {\mathbb{R}}^{1\times W_{i}\times H_{i}}$. The attention can be formulated as follows:


(4) $$\begin{array}{l} Att_{i}=sigmoid\left(\sigma \left(f_{i_{1}}\left(C_{i},w_{i_{1}}\right)⊗f_{i_{2}}\left(C_{i},w_{i_{2}}\right)\right)\right) \end{array}$$

where *f*_*i*_*j*__(·, ·) denotes an atrous convolution function, ⊗ represents element-wise multiplication, σ means a 1 × 1 convolution, and *sigmoid* indicates the Sigmoid activation function, $Att_{i}\in {\mathbb{R}}^{1\times W_{i}\times H_{i}}$ is the attention generate from the contour. In this process, the atrous convolutions *f* are employed to mine spatial information from contour. During the experiments, in our experience, the kernel sizes of both *f*_*i*_1__ and *f*_*i*_2__ are set to 15, with a dilation rate of 3, and they do not share weights.

To emphasize the feature of object, we formulate the attention weighted map $Att_{i}^{\prime }$ as 1−*Att*_*i*_. Then the enhanced feature map $P_{i}^{\prime }\in {\mathbb{R}}^{N\times W_{i}\times H_{i}}$ can be presented as:


(5) $$\begin{array}{l} P_{i}^{\prime }=P_{i}⊗Att_{i}^{\prime }⊕P_{i} \end{array}$$

where ⊕ means element-wise sum.

Inspired by Tan et al. (2020), an inverse aggregation method is designed to utilize features in low levels assistant for large instance identification. We designed the structure to allow low-level features to provide detailed information for nearby high-level features, which implemented by resizing the low-level feature map $P_{i-1}^{\prime }\in {\mathbb{R}}^{N\times W_{i-1}\times H_{i-1}}$ to the same scale as near high-level feature map $P_{i}^{\prime }\in {\mathbb{R}}^{N\times W_{i}\times H_{i}}$ and employing the element-wise sum. The *i*-th feature $P_{i}^{\prime \prime }\in {\mathbb{R}}^{N\times W_{i}\times H_{i}}$ for segmentation and detection can be generated as:


(6) $$\begin{array}{l} P_{i}^{\prime \prime }=P_{i}^{\prime }⊕\delta \left(P_{i-1}^{\prime }\right) \end{array}$$

where δ means down-sampling. It is worth noting that since $P_{2}^{\prime \prime }$ has no lower-level features, in practice $P_{2}^{\prime \prime }$ and $P_{2}^{\prime }$ are the same. At this point, enhancement of features based on contours is complete.

### 3.3 Instance segmentation head

Following the Mask R-CNN (He et al., 2017), our instance segmentation head produces bounding box regression, classification, and segmentation mask from $P_{2}^{\prime \prime }-P_{5}^{\prime \prime }$. The purpose of this head is to provide pixel-level annotations for each instance. The loss function *L*_*ins*_ is defined as follows:


(7) $$\begin{array}{l} L_{ins}=L_{cls}+L_{bbox}+L_{mask} \end{array}$$

where *L*_*cls*_ is the is the classification loss, *L*_*bbox*_ is the object bounding-box regression loss, and *L*_*mask*_ is the average binary cross-entropy loss for mask prediction.

### 3.4 Semantic segmentation head

For semantic segmentation head, we stack two 3 × 3 deformable convolution layers following SFMM features. Similar to PSPNet (Zhao et al., 2017), we first scale the features of different sizes to the same scale to achieve fusion of different granularity. Then, the semantic presentations are obtained by concatenating these scaled features. For this head, we choose the standard cross entropy in semantic segmentation as the loss function, denoted as *L*_*seg*_.

During training, the total loss *L*_*total*_ is formulated as:


(8) $$\begin{array}{l} L_{total}=L_{C}+L_{ins}+L_{seg} \end{array}$$

## 4 Experiments

In this section, our CCPSNet is evaluated on Cityscapes (Cordts et al., 2016) and Microsoft COCO (Lin et al., 2014) datasets. We present the experimental results on these datasets and compare with them the state-of-the-art models based on Mask R-CNN. The ablation studies and robustness analysis are presented at last.

### 4.1 Datasets and metrics

#### 4.1.1 Cityscapes

This dataset focuses on understanding urban streets scenes. It is composed of 2,975 training images, 500 validation images, and 1,525 test images. All these 5,000 images are with fine annotations. There are another 20,000 images with coarse annotations, which are not utilized in our experiment. This dataset has a total of 19 categories, of which 8 are things and the remaining 11 are stuff. In this dataset, we use 4 RTX 2080Ti GPUs to train our model, and trained in a batch size of 1 per GPU, learning rate of 0.02 and weight decay of 1*e*^−4^ for 48,000 steps in total and decay the learning rate by a factor of 0.1 at step 36,000.

#### 4.1.2 COCO

This dataset contains a large number of natural images, comprising both indoor and outdoor scenes. The distribution between images is also inconsistent, making it challenging for algorithms to learn good results. It contains 140,000 images with 115,000 training images, 5,000 validation images, 20,000 test-dev images, and 20,000 test images. There are 80 thing categories and 53 stuff categories. We only rely on the train set with no extra data, presenting the results on the validation set for comparison. In this dataset, we use 8 RTX 2080Ti GPUs to train our model, and trained in a batch size of 1 per GPU, learning rate of 0.01 and weight decay of 1*e*^−4^ for 48,000 steps in total and decay the learning rate by a factor of 0.1 at step 240,000 and 32,000.

#### 4.1.3 Evaluation metrics

Following Kirillov et al. (2019b), the Panoptic Quality (PQ) is adopted as evaluation metrics:


$$\begin{array}{l} PQ=\underset{segmentation\;quality\left(SQ\right)}{\underset{︸}{\frac{\underset{\left(p,g\right)\in TP}{\sum }IoU\left(p,g\right)}{\left|TP\right|}}}\times \underset{recognition\;quality\left(RQ\right)}{\underset{︸}{\frac{\left|TP\right|}{\left|TP|+\frac{1}{2}|FP|+\frac{1}{2}|FN\right|}}}, \end{array}$$

According to the formula, PQ is determined by multiplying SQ and RQ, combining the evaluation of semantic segmentation and instance segmentation. In the formula, *IoU*(*p, g*) means the intersection-over-union between predicted object *p* and ground truth *g*. *TP* (True Positives) represents matched pairs of segments, and *FP* (False Positive) means unmatched pairs of segments, and *FN* (False Negatives) means unmatched ground truth segments. When the value of *IoU* is >0.5, it is considered a positive match. Note that *PQ*, *PQ*^*Th*^, and *PQ*^*St*^ refer to the *PQ* values averaged across all classes, thing classes and stuff classes.

### 4.2 Comparsion to state-of-the-art

We compare our proposed network with other state-of-the-art methods on Cityscapes (Cordts et al., 2016) val set and MS-COCO (Lin et al., 2014).

#### 4.2.1 Cityscapes

**Figure 5:**
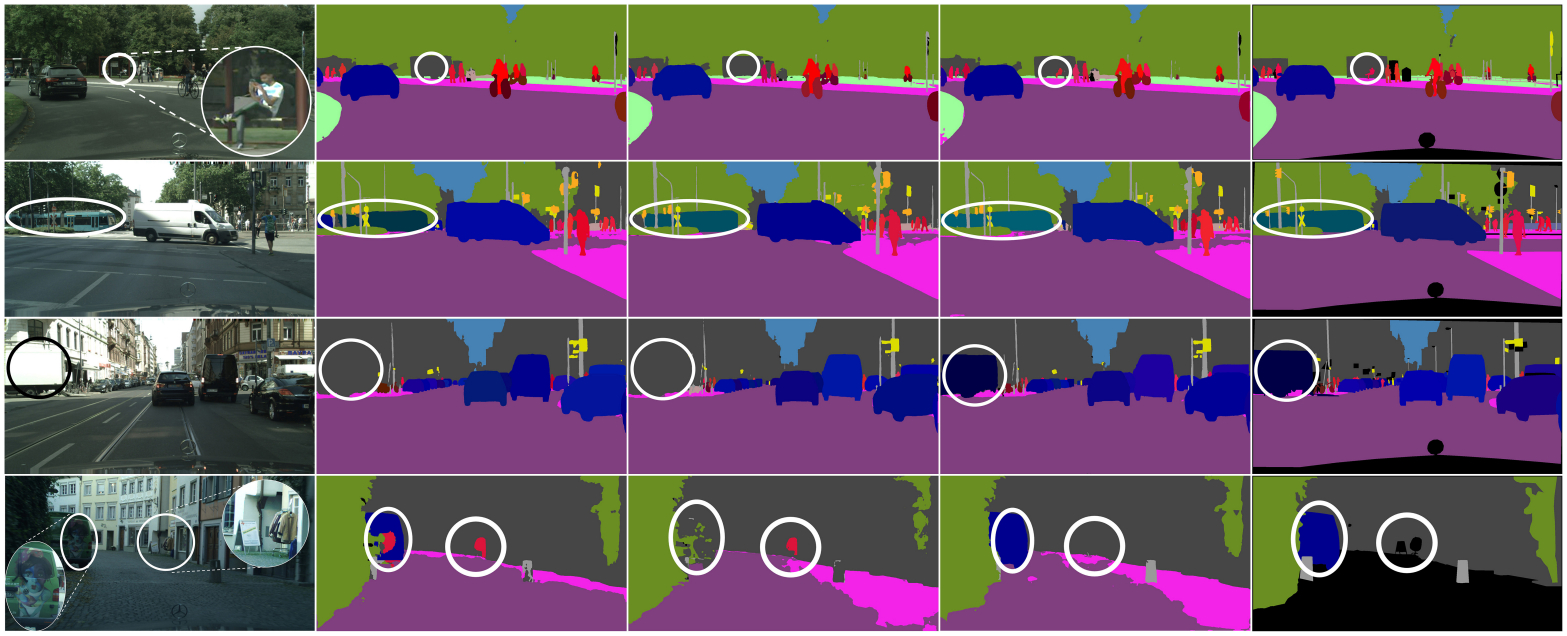
Visual examples of panoptic segmentation on Cityscapes. From left to right are input images, predicted results from UPSNet, CAPSNet, CCPSNet (ours), and ground truth.

As shown in Table 1, the proposed CCPSNet of ResNet-50 is used to realize 60.5% and CCPSNet of ResNet-101 is used to realize 61.2% which is improved compared with similar methods with same backbone. Figure 5 presents some visual examples of our algorithm on Cityscapes. The first row shows that the problem of small target objects, such as cyclists, which is difficult to distinguish due to lighting problems is alleviated by the feature cascade fusion module proposed by CCPSNet. The second and third rows show that when there is occlusion between different objects, contour perception in CCPSNet can solve this problem very well. The fourth row shows that through contour perception and contour feature enhancement, CCPSNet can effectively detect ambiguous scenes such as the left side of the vehicle painted with a face pattern and the right side of the street hanging a row of clothes.

**Table 1:** Comparsion with other methods on Cityscapes val sets.

**Method**	***PQ***	***PQ* ^*Th*^**	***PQ* ^*St*^**
**Backbone: ResNet-50 (He et al.**, **2016****)**			
EfficientPS (Mohan and Valada, 2021)	60.3	55.3	63.9
Panoptic-FPN (Kirillov et al., 2019a)	57.7	51.6	62.2
UPSNet (Xiong et al., 2019)	59.1	54.1	62.7
AUNet (Li et al., 2019)	56.4	52.7	59.0
CAPSNet (Xu et al., 2021)	60.0	55.7	63.1
YOSO (Hu et al., 2023)	59.7	51.0	**66.1**
LPSNet (Hong et al., 2021)	59.7	54.0	63.9
SE-PSNet (Chang et al., 2023)	60.0	55.9	62.9
CCPSNet (Ours)	**60.5**	**56.9**	63.1
**Backbone: ResNet-101 (He et al.**, **2016****)**			
EfficientPS (Mohan and Valada, 2021)	61.1	56.5	**64.2**
Panoptic-FPN (Kirillov et al., 2019a)	58.1	52.0	62.5
AUNet (Li et al., 2019)	59.0	54.8	62.1
AdaptIS (Sofiiuk et al., 2019)	60.6	**57.5**	62.6
CCPSNet (Ours)	**61.2**	57.1	64.1

The bold value means the best result in the column.

We evaluate the segmentation results on specific categories in Cityscapes, where the object size is smaller than 32 pixels × 32 pixels. The results based on ResNet-50 are reported in Table 2. It can be observed that our proposed algorithm indeed improves the performance for small objects, thanks to contour-guided multi-scale feature enhancement stream.

**Table 2:** Accuracy of small object on CityScapes val set.

**Method**	***PQ***	***SQ***	***RQ***
UPSNet (Xiong et al., 2019)	50.26	71.70	70.13
CAPSNet (Xu et al., 2021)	51.01	**72.70**	70.19
CCPSNet (Ours)	**51.26**	72.53	**70.70**

The bold value means the best result in the column.

#### 4.2.2 COCO

In addition to verification in outdoor driving scenarios, we further demonstrated the universality of our method on the COCO dataset, which includes various indoor and outdoor scenes. As shown in Table 3, we compare with similar methods with same backbone. The proposed CCPSNet achieves the PQ 43.2% and the PQ 43.5% with backbone ResNet-101.

**Table 3:** Comparsion with other methods on COCO val sets.

**Method**	*PQ*	*PQ* ^*Th*^	*PQ* ^*St*^
**Backbone: ResNet-50 (He et al.**, **2016****)**			
UPSNet (Xiong et al., 2019)	42.5	48.5	33.4
TASCNet (Li et al., 2018)	40.7	47.0	31.0
SpatialFlow (Chen Q. et al., 2020)	42.9	49.5	33.0
Panoptic FPN (Kirillov et al., 2019a)	39.0	45.9	28.7
OANet (Liu et al., 2019)	41.3	**50.4**	27.7
JSIS-Net (De Geus et al., 2018)	26.9	29.3	23.3
AUNet (Li et al., 2019)	39.6	49.1	25.2
AdaptIS (Sofiiuk et al., 2019)	35.9	40.3	29.3
CIAE (Gao et al., 2021)	40.2	45.3	32.3
SOLO V2 (Wang X. et al., 2020)	42.1	49.6	30.7
OCFusion (Lazarow et al., 2020)	41.3	49.4	29.0
LPSNet (Hong et al., 2021)	39.1	43.9	30.1
IDNet (Lin et al., 2023)	42.1	47.5	33.9
CCPSNet (Ours)	**43.0**	49.2	**33.6**
**Backbone: ResNet-101 (He et al.**, **2016****)**			
Panoptic-FPN (Kirillov et al., 2019a)	40.3	47.5	29.5
AdaptIS (Sofiiuk et al., 2019)	37.0	41.8	29.9
OCFusion (Lazarow et al., 2020)	43.0	**51.1**	30.7
SSAP (Gao et al., 2019)	36.9	40.1	32.0
CCPSNet (Ours)	**43.5**	49.9	**33.8**

The bold value means the best result in the column.

**Figure 6:**
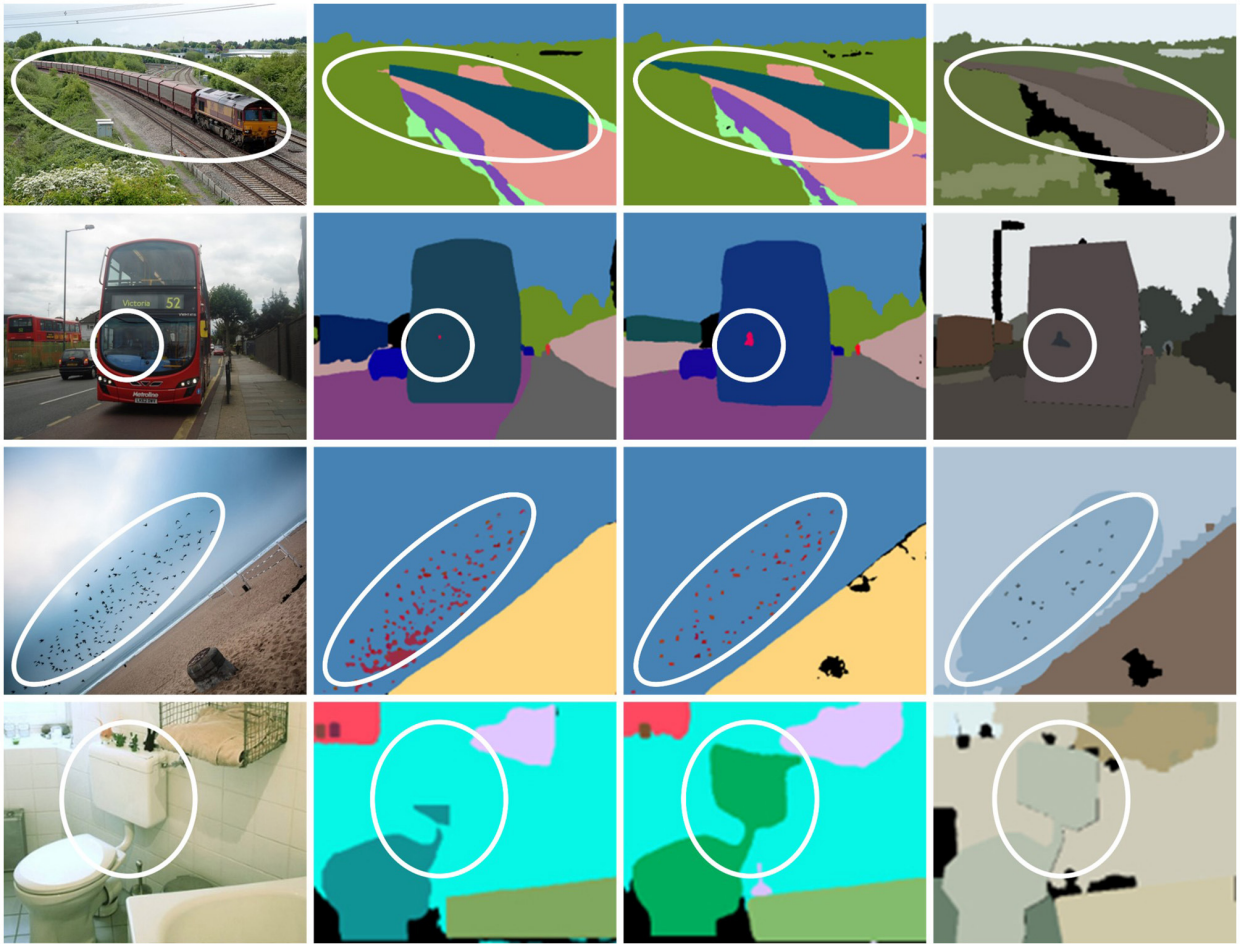
Visual examples of panoptic segmentation on COCO. From left to right are input images, predicted results from UPSNet, CCPSNet (ours), and ground truth.

Figure 6 presents some visual examples of our algorithm on MS-COCO. The first row presented is similar to the one in CityScapes in its ability to detect extra-long targets such as trains, and the distant train body is well preserved. The second row is mainly for the detection of bus drivers, and the information of the characters is better retained. The third row focuses on the detection of small targets of bird flocks. Unlike UPSNet, CCPSNet still retains the good individual characteristics of flying birds for a large number of small targets and does not show a large number of block-like structures, and there are relatively independent characteristics among flying birds from the original figure. The fourth row shows the structure of the flush toilet, which is well preserved in CCPSNet, even if the structure of the drain is clearly visible.

### 4.3 Ablation studies

To demonstrate the effectiveness of each component in our network, we conduct related ablation experiments. Table 4 shows the quantitative ablative analysis, where empty cells mean the corresponding components are not adopted.

**Table 4:** Results of ablation experiments on CityScapes val set.

**Cascade contour detection stream**		**Contour-guided multi-scale feature enhancement stream**		**PQ**	**PQ^*th*^**	**PQ^*st*^**	**SQ**	**RQ**
**Cascade structure**	**CRSPM**	**SFMM**	**Inverse aggregation**					
				59.1	54.1	62.7	80.1	72.4
✓				59.6	54.9	63.0	80.0	73.2
✓	✓			59.8	55.2	**63.1**	80.0	73.4
✓	✓	✓		60.1	55.9	**63.1**	80.2	73.6
✓	✓	✓	✓	**60.5**	**56.9**	**63.1**	**80.3**	**74.1**

The bold value means the best result in the column.

In Table 4, the first row shows the results of the baseline without any innovative design. The second row represents the experimental results for the cascade contour stream without CRSPM we designed. This outcome demonstrates that the introduction of contours effectively enhances the scene perception capabilities, particularly in terms of the overall evaluation metric PQ and the object recognition metric RQ. Compared to the second row, the third row exhibits the effectiveness of CRSPM. As can be seen from the results, CRSPM further improves performance. We believe that this performance enhancement is primarily due to the following reasons. The inclusion of the contour detection head can encourage base feature extractor to focus on learning structual features, and channel regulation structural perception module employ a GAP and 1 × 1 convolution to re-weight the channels, selecting those sensitive to contour perception and allowing them to cascade participate in the perception process. We utilize the same global average pooling operation as in Condori and Bruno (2021) to preserve the texture information. This further indicates that introducing the contour recognition function of the visual cortex of the brain in the task of scene recognition can effectively improve the performance of performance performance of the network. The cascaded panoptic segmentation contour branch proposed by CCPSNet can perceive the contour more finely.

To validate the effectiveness of contour-guided multi-scale feature enhancement stream, we conducted experimental verification of structural-aware feature modulation module(SFMM) and inverse aggregation based on the third-row model. It is worth noting that the introduction of inverse aggregation improves PQ by 1%, indicating that this design can indeed help improve instance detection. It can be seen that the contour-guided multi-scale feature enhancement stream brings a 0.7% performance gain to the overall network metrics, including a 1.7% performance gain to the foreground panoptic segmentation *PQ*^*th*^ and a 0.3% gain to the background panoptic segmentation SQ. This is mainly due to the introduction of contour information and the enhancement of the features with more detailed information through bottom-up feature cascading, which also helps semantic segmentation.

### 4.4 Robustness analysis

**Figure 7:**
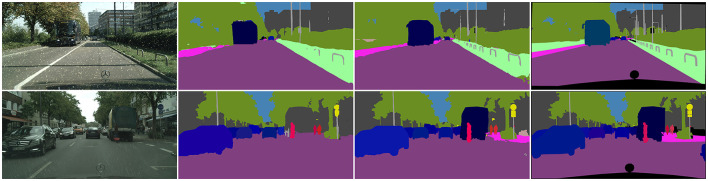
Visualization examples of panoptic segmentation on data augmentation Cityscapes. From left to right are input images, predicted results from UPSNet, CCPSNet (ours), and ground truth.

When a robot perceives its environment in the real world, it encounters various types of distortion at each stage of visual signal acquisition, compression and transmission. Additionally, it may face challenges such as rainy days and camera dirt, which can affect image quality and subsequently impact the algorithm's performance. Many studies (Zhai and Min, 2020; Min et al., 2024) have shown that image quality is essential for artificial intelligence, and low-quality input will impact the algorithm's performance. In this regard, we conduct experimental analysis on the robustness of our method to the input image quality. We applied image processing on the CityScapes dataset to verify the robustness of our network. By calling the imgaug (Jung et al., 2020) library functions, rain and noise were incorporated into the images to simulate challenging conditions such as rainy scenes and dirty cameras, which are commonly encountered by robots during operation. We evaluated the model trained on the original Cityscapes dataset directly on new data without additional training to test the algorithm's robustness. The experimental results, presented in Table 5, compare the performance of the proposed method and the UPSNet in these scenarios. In the case of simulated rainy days and dirty cameras, our algorithm achieved PQ scores of 50.4% and 39.7%, respectively. The results demonstrate varying degrees of performance degradation compared to the original dataset, but our proposed algorithm continues to outperform in these complex scenarios. This is mainly caused by the different impacts of noise on the image. As shown in Figure 7, it can be seen that the rain image has little change compared with the original image, but the simulated dirty image has a large change. In the top row picture, it is evident that our algorithm still has a strong ability to perceive contours in complex scenes. In the bottom row picture, it is evident that the introduction of contours has improved our performance in dealing with large textureless areas. This demonstrates the promising robustness of the proposed CCPSNet in challenging scenarios faced by robots.

**Table 5:** Accuracy on noise CityScapes val set.

**Sense**	**Method**	***PQ***	***SQ***	***RQ***
Dirtiness	UPSNet (Xiong et al., 2019)	37.9	69.9	48.8
	CCPSNet (Ours)	**39.7**	**74.6**	**51.0**
Rainy	UPSNet (Xiong et al., 2019)	49.5	76.9	62.5
	CCPSNet (Ours)	**50.4**	**78.1**	**62.9**

The bold value means the best result in the column.

## 5 Conclusions

In this paper, we introduced a novel panoptic segmentation algorithm that relies on panoptic segmentation contour guidance. Our approach proposes a new cascade contour detection stream that coarse-to-fine extracts scene structural information. We also developed a contour-guided multi-scale feature enhancement stream that fully utilizes the extracted contours. In addition, our feature inverse aggregation structure enables a bi-directional flow of features to achieve perceptual enhancement of small objects. Finally, the experimental results on Cityscapes and COCO show that our algorithm is highly competitive with similar algorithms. We verify the robustness of the proposed algorithm in complex environments by simulating rain and camera dirtiness with data augmentation. In future work, we plan to extend this idea to unsupervised segmentation tasks.

## Data Availability

The original contributions presented in the study are included in the article/supplementary material, further inquiries can be directed to the corresponding author.

## References

[B1] Alazeb A. Chughtai B. R. Al Mudawi N. AlQahtani Y. Alonazi M. Aljuaid H.. (2024). Remote intelligent perception system for multi-object detection. Front. Neurorobot. 18:1398703. 10.3389/fnbot.2024.1398703 38831877 PMC11144911

[B2] Carion N. Massa F. Synnaeve G. Usunier N. Kirillov A. Zagoruyko S. (2020). “End-to-end object detection with transformers,” in European conference on computer vision (Springer), 213–229. 10.1007/978-3-030-58452-8_13

[B3] Chang S.-E. Chen Y. Yang Y.-C. Lin E.-T. Hsiao P.-Y. Fu L.-C. (2023). Se-psnet: Silhouette-based enhancement feature for panoptic segmentation network. J. Vis. Commun. Image Represent. 90:103736. 10.1016/j.jvcir.2022.103736

[B4] Chen L.-C. Papandreou G. Kokkinos I. Murphy K. Yuille A. L. (2017). Deeplab: semantic image segmentation with deep convolutional nets, atrous convolution, and fully connected crfs. IEEE Trans. Pattern Anal. Mach. Intell. 40, 834–848. 10.1109/TPAMI.2017.2699184 28463186

[B5] Chen Q. Cheng A. He X. Wang P. Cheng J. (2020). Spatialflow: bridging all tasks for panoptic segmentation. IEEE Trans. Circ. Syst. Video Technol. 31, 2288–2300. 10.1109/TCSVT.2020.3020257

[B6] Chen Y. Lin G. Li S. Bourahla O. Wu Y. Wang F.. (2020). “Banet: bidirectional aggregation network with occlusion handling for panoptic segmentation,” in Proceedings of the IEEE/CVF Conference on Computer Vision and Pattern Recognition, 3793–3802. 10.1109/CVPR42600.2020.00385

[B7] Cheng B. Collins M. D. Zhu Y. Liu T. Huang T. S. Adam H.. (2020). “Panoptic-deeplab: A simple, strong, and fast baseline for bottom-up panoptic segmentation,” in Proceedings of the IEEE/CVF Conference on Computer Vision and Pattern Recognition, 12475–12485. 10.1109/CVPR42600.2020.01249

[B8] Condori R. H. Bruno O. M. (2021). Analysis of activation maps through global pooling measurements for texture classification. Inf. Sci. 555, 260–279. 10.1016/j.ins.2020.09.058

[B9] Cordts M. Omran M. Ramos S. Rehfeld T. Enzweiler M. Benenson R.. (2016). “The cityscapes dataset for semantic urban scene understanding,” in Proceedings of the IEEE Conference on Computer Vision and Pattern Recognition, 3213–3223. 10.1109/CVPR.2016.35032191886

[B10] De Geus D. Meletis P. Dubbelman G. (2018). Panoptic segmentation with a joint semantic and instance segmentation network. arXiv preprint arXiv:1809.02110.

[B11] Gao N. Shan Y. Wang Y. Zhao X. Yu Y. Yang M.. (2019). “Ssap: single-shot instance segmentation with affinity pyramid,” in Proceedings of the IEEE/CVF International Conference on Computer Vision, 642–651. 10.1109/ICCV.2019.00073

[B12] Gao N. Shan Y. Zhao X. Huang K. (2021). Learning category-and instance-aware pixel embedding for fast panoptic segmentation. IEEE Trans. Image Proc. 30, 6013–6023. 10.1109/TIP.2021.3090522 34181542

[B13] He K. Gkioxari G. Dollár P. Girshick R. (2017). “Mask R-CNN,” in Proceedings of the IEEE International Conference on Computer Vision, 2961–2969. 10.1109/ICCV.2017.322

[B14] He K. Zhang X. Ren S. Sun J. (2016). “Deep residual learning for image recognition,” in Proceedings of the IEEE Conference on Computer Vision and Pattern Recognition, 770–778. 10.1109/CVPR.2016.90

[B15] Hong W. Guo Q. Zhang W. Chen J. Chu W. (2021). “Lpsnet: a lightweight solution for fast panoptic segmentation,” in Proceedings of the IEEE/CVF Conference on Computer Vision and Pattern Recognition, 16746–16754. 10.1109/CVPR46437.2021.01647

[B16] Hu J. Huang L. Ren T. Zhang S. Ji R. Cao L. (2023). “You only segment once: towards real-time panoptic segmentation,” in Proceedings of the IEEE/CVF Conference on Computer Vision and Pattern Recognition, 17819–17829. 10.1109/CVPR52729.2023.01709

[B17] Jung A. B. Wada K. Crall J. Tanaka S. Graving J. Reinders C.. (2020). Imgaug. Available at: https://github.com/aleju/imgaug (accessed February 01, 2020).

[B18] Kirillov A. Girshick R. He K. Dollár P. (2019a). “Panoptic feature pyramid networks,” in Proceedings of the IEEE/CVF Conference on Computer Vision and Pattern Recognition, 6399–6408. 10.1109/CVPR.2019.00656

[B19] Kirillov A. He K. Girshick R. Rother C. Dollár P. (2019b). “Panoptic segmentation,” in Proceedings of the IEEE/CVF Conference on Computer Vision and Pattern Recognition, 9404–9413. 10.1109/CVPR.2019.00963

[B20] Lazarow J. Lee K. Shi K. Tu Z. (2020). “Learning instance occlusion for panoptic segmentation,” in Proceedings of the IEEE/CVF Conference on Computer Vision and Pattern Recognition, 10720–10729. 10.1109/CVPR42600.2020.01073

[B21] Li J. Raventos A. Bhargava A. Tagawa T. Gaidon A. (2018). Learning to fuse things and stuff. arXiv preprint arXiv:1812.01192.

[B22] Li X. Li X. Zhang L. Cheng G. Shi J. Lin Z.. (2020). “Improving semantic segmentation via decoupled body and edge supervision,” in Computer Vision-ECCV 2020: 16th European Conference, Glasgow, UK, August 23-28, 2020, Proceedings, Part XVII 16 (Springer), 435–452. 10.1007/978-3-030-58520-4_26

[B23] Li Y. Chen X. Zhu Z. Xie L. Huang G. Du D.. (2019). “Attention-guided unified network for panoptic segmentation,” in Proceedings of the IEEE Conference on Computer Vision and Pattern Recognition, 7026–7035. 10.1109/CVPR.2019.00719

[B24] Lin G. Li S. Chen Y. Li X. (2023). IDNet: information decomposition network for fast panoptic segmentation. IEEE Trans. Image Proc. 33, 1487–1496. 10.1109/TIP.2023.3234499 37037237

[B25] Lin T.-Y. Maire M. Belongie S. Hays J. Perona P. Ramanan D.. (2014). “Microsoft coco: common objects in context,” in Computer Vision-ECCV 2014: 13th European Conference, Zurich, Switzerland, September 6-12, 2014, Proceedings, Part V 13 (Springer), 740–755. 10.1007/978-3-319-10602-1_48

[B26] Liu H. Peng C. Yu C. Wang J. Liu X. Yu G.. (2019). “An end-to-end network for panoptic segmentation,” in Proceedings of the IEEE/CVF Conference on Computer Vision and Pattern Recognition, 6172–6181. 10.1109/CVPR.2019.00633

[B27] Liu T. Stathaki T. (2018). Faster R-cnn for robust pedestrian detection using semantic segmentation network. Front. Neurorobot. 12:64. 10.3389/fnbot.2018.00064 30344486 PMC6182048

[B28] Min X. Duan H. Sun W. Zhu Y. Zhai G. (2024). Perceptual video quality assessment: a survey. arXiv preprint arXiv:2402.03413.

[B29] Min X. Zhai G. Gu K. Yang X. (2016). Fixation prediction through multimodal analysis. ACM Trans. Multim. Comput. Commun. Applic. 13, 1–23. 10.1145/2996463

[B30] Min X. Zhai G. Zhou J. Zhang X.-P. Yang X. Guan X. (2020). A multimodal saliency model for videos with high audio-visual correspondence. IEEE Trans. Image Proc. 29, 3805–3819. 10.1109/TIP.2020.2966082 31976898

[B31] Mohan R. Valada A. (2021). Efficientps: efficient panoptic segmentation. Int. J. Comput. Vis. 129, 1551–1579. 10.1007/s11263-021-01445-z

[B32] Sofiiuk K. Barinova O. Konushin A. (2019). “Adaptis: adaptive instance selection network,” in Proceedings of the IEEE/CVF international conference on computer vision, 7355–7363. 10.1109/ICCV.2019.00745

[B33] Sun B. Kuen J. Lin Z. Mordohai P. Chen S. (2023). “PRN: panoptic refinement network,” in Proceedings of the IEEE/CVF Winter Conference on Applications of Computer Vision, 3963–3973. 10.1109/WACV56688.2023.00395

[B34] Takikawa T. Acuna D. Jampani V. Fidler S. (2019). “Gated-SCNN: gated shape cnns for semantic segmentation,” in Proceedings of the IEEE/CVF International Conference on Computer Vision, 5229–5238. 10.1109/ICCV.2019.00533

[B35] Tan M. Pang R. Le Q. V. (2020). “Efficientdet: scalable and efficient object detection,” in Proceedings of the IEEE/CVF Conference on Computer Vision and Pattern Recognition, 10781–10790. 10.1109/CVPR42600.2020.01079

[B36] Wang H. Zhu Y. Adam H. Yuille A. Chen L.-C. (2021). “Max-deeplab: end-to-end panoptic segmentation with mask transformers,” in Proceedings of the IEEE/CVF Conference on Computer Vision and Pattern Recognition, 5463–5474. 10.1109/CVPR46437.2021.00542

[B37] Wang H. Zhu Y. Green B. Adam H. Yuille A. Chen L.-C. (2020). “Axial-deeplab: stand-alone axial-attention for panoptic segmentation,” in European Conference on Computer Vision (Springer), 108–126. 10.1007/978-3-030-58548-8_7

[B38] Wang X. Zhang R. Kong T. Li L. Shen C. (2020). Solov2: Dynamic and fast instance segmentation. Adv. Neural Inf. Process. Syst. 33, 17721–17732.

[B39] Xie S. Tu Z. (2015). “Holistically-nested edge detection,” in Proceedings of the IEEE International Conference on Computer Vision, 1395–1403. 10.1109/ICCV.2015.164

[B40] Xiong Y. Liao R. Zhao H. Hu R. Bai M. Yumer E.. (2019). “Upsnet: a unified panoptic segmentation network,” in Proceedings of the IEEE/CVF Conference on Computer Vision and Pattern Recognition, 8818–8826. 10.1109/CVPR.2019.00902

[B41] Xu Y. Zhu D. Zhang G. Shi W. Li J. Zhang X. (2021). “Contour-aware panoptic segmentation network,” in Pattern Recognition and Computer Vision: 4th Chinese Conference, PRCV 2021, Beijing, China, October 29-November 1, 2021, Proceedings, Part II, 79–90. 10.1007/978-3-030-88007-1_7

[B42] Yang L. Lei W. Zhang W. Ye T. (2023). Dual-flow network with attention for autonomous driving. Front. Neurorobot. 16:978225. 10.3389/fnbot.2022.978225 36699946 PMC9868693

[B43] Yang T.-J. Collins M. D. Zhu Y. Hwang J.-J. Liu T. Zhang X.. (2019). Deeperlab: single-shot image parser. arXiv preprint arXiv:1902.05093.

[B44] Ye X. Gao L. Chen J. Lei M. (2023). Based on cross-scale fusion attention mechanism network for semantic segmentation for street scenes. Front. Neurorobot. 17:1204418. 10.3389/fnbot.2023.1204418 37719330 PMC10501793

[B45] Yu Q. Wang H. Kim D. Qiao S. Collins M. Zhu Y.. (2022a). “CMT-deeplab: clustering mask transformers for panoptic segmentation,” in Proceedings of the IEEE/CVF Conference on Computer Vision and Pattern Recognition, 2560–2570. 10.1109/CVPR52688.2022.00259

[B46] Yu Q. Wang H. Qiao S. Collins M. Zhu Y. Adam H.. (2022b). “K-means mask transformer,” in European Conference on Computer Vision (Springer), 288–307. 10.1007/978-3-031-19818-2_17

[B47] Zhai G. Min X. (2020). Perceptual image quality assessment: a survey. Sci. China Inform. Sci. 63:1–52. 10.1007/s11432-019-2757-1

[B48] Zhang C. Xu F. Wu C. Xu C. (2022). A lightweight multi-dimension dynamic convolutional network for real-time semantic segmentation. Front. Neurorobot. 16:1075520. 10.3389/fnbot.2022.1075520 36590086 PMC9797588

[B49] Zhang C. Xu F. Wu C. Xu C. (2023). Rethinking 1D convolution for lightweight semantic segmentation. Front. Neurorobot. 17:1119231. 10.3389/fnbot.2023.1119231 36845064 PMC9947531

[B50] Zhao H. Shi J. Qi X. Wang X. Jia J. (2017). “Pyramid scene parsing network,” in Proceedings of the IEEE Conference on Computer Vision and Pattern Recognition, 2881–2890. 10.1109/CVPR.2017.660

[B51] Zhen M. Wang J. Zhou L. Li S. Shen T. Shang J.. (2020). “Joint semantic segmentation and boundary detection using iterative pyramid contexts,” in Proceedings of the IEEE/CVF Conference on Computer Vision and Pattern Recognition, 13666–13675. 10.1109/CVPR42600.2020.01368

[B52] Zhou H. Friedman H. S. Von Der Heydt R. (2000). Coding of border ownership in monkey visual cortex. J. Neurosci. 20, 6594–6611. 10.1523/JNEUROSCI.20-17-06594.2000 10964965 PMC4784717

